# Because You Watched: How Do Streaming Services’ Recommender Systems Influence Aesthetic Choice?

**DOI:** 10.3390/bs15111544

**Published:** 2025-11-13

**Authors:** Harrison E. Chapman, Anna Abraham

**Affiliations:** 1 Department of Educational Psychology, Mary Frances Early College of Education, University of Georgia, Aderhold Hall, 110 Carlton Street, Athens, GA 30602, USA; anna.abraham@uga.edu; 2 Torrance Center for Creativity, Mary Frances Early College of Education, University of Georgia, Aderhold Hall, 110 Carlton Street, Athens, GA 30602, USA

**Keywords:** entertainment preferences, aesthetic experience, media psychology, well-being, decision-making, algorithmic recommender systems, audiovisual media

## Abstract

To support users’ media selection, streaming services increasingly rely on algorithmic recommender systems that provide personalized media curation based on various sources of user information (e.g., previously watched content). The utilization of recommender systems has led to concerns over how such systems might influence an individual’s aesthetic choices. Recommender systems and increased accessibility to media content have, after all, changed the way users engage with entertainment media. This paper examines the relationship between recommender systems and individuals’ aesthetic choices by exploring literature from disparate fields such as modern media and algorithms, aesthetic choice, entertainment preferences, and well-being. We explore the notion that aesthetic choice can be considered a “low-stake” decision process and is part of a fulfilling aesthetic life, influencing the individual through incremental and cumulative decisions. The paper provides considerations for future directions for exploration as this burgeoning field leaves us with more questions than answers.

## 1. Introduction

Modern technology has enabled an omnipresent daily choice of audiovisual entertainment consumption. Whether on a phone, tablet, or television, a diverse selection of entertainment media exists, and people spend a significant amount of time-consuming audiovisual content. Over a relatively short period of time, we have witnessed staggering developments in media access and selection. Movie consumers had highly restricted options of when, where, and how often they watched movies. In 2007, Netflix began streaming media content directly to people’s electronic devices. Since then, a wide variety of on-demand subscription video services have surfaced, providing an unprecedented amount of choice for the average consumer.

At the same time, algorithmic recommender systems have blossomed and are heavily utilized by media streaming companies to support user engagement, as we have endless options to choose from at our fingertips. The influence of the accessibility and curation of media consumption by recommender systems on our aesthetic choices is an under-researched area, leaving us with more questions than answers. For example, how much autonomy of aesthetic choice do consumers have? How have our preferences for media consumption changed with the accessibility of media selection available to us? How are our aesthetic sensibilities changed when we engage with a recommendation from a human compared to an algorithmic recommender?

This paper explores the relationship between algorithmic recommender systems and people’s aesthetic choices in audiovisual entertainment by examining a selection of literature from disparate fields, such as philosophy, psychology, and human-recommender interaction^1^. These domains provide an opportunity to explore an understudied area through interdisciplinary approaches that overlap in terms of topical focus yet remain largely disconnected in terms of discourse. As the evidence on hand is too limited to allow for a systematic review, this article presents a selective overview of bringing these different disciplines together. The paper is organized to cover the following themes: (a) modern media consumption and algorithms, (b) aesthetics and choice, (c) entertainment preferences and well-being, and (d) implications and future directions.

## 2. Modern Media Consumption and Algorithms

Several modern streaming providers (e.g., Disney+, Apple TV, Peacock, and HBO Max) launched their services between November 2019 and May 2020 (Pajkovic, 2022), and the increased availability of streaming platforms has forced companies to compete for consumer retention and selection. The growth of streaming content has led to a saturation of over 76,000 different types of alt-genres to describe entertainment categories (Madrigal, 2014). The differentiation of genres attempts to allow viewers the opportunity to have a “personalized” viewing experience. Personalization on streaming platforms is accomplished typically through an algorithm matching the “right” type of entertainment selection to the user via recommendations of what to watch. As Netflix highlights, “internet TV is about choice… but humans are surprisingly bad at choosing between many options, quickly getting overwhelmed” (Gomez-Uribe & Hunt, 2016, p. 2). Therefore, the underlying idea is that content must be curated, and in streaming shows and movies, machine-learning-algorithmic recommender systems most often direct the curation of content.

Gillespie (2014) suggests that algorithms facilitate and manage the information-seeking process and flow, and connection to participation. Broadly speaking, algorithms reflect encoded computational procedures of input values that are transformed into output data (Gillespie, 2014; Varela & Kaun, 2019). Algorithms are designed and specified instructions for a given task, but in the case of machine learning, the algorithms derive the final model from the data itself with the goal of prediction (Kearns & Roth, 2019). Machine learning is a type of artificial intelligence where computers simulate “human learning” through knowledge acquisition from real-world data and then improve performance from their knowledge (Portugal et al., 2018, p. 206). As Vatsal (2022, para. 1) states, a machine-learning “recommender system is a system which predicts ratings a user might give to a specific item. These predictions will then be ranked and returned back to the user.” In video streaming services, the goal is to predict media content that the user would watch to support user decision-making processes. As the goal is to keep users engaged, a prediction is accurate if a person picks and watches the content provided by the recommender system.

Companies utilize several different recommender system models, often referred to as filtering. Collaborative filtering (CF) is one of the primary techniques for movie recommender systems, which offer personalized recommendations through a model- or memory-based system (Thakker et al., 2021). Additional types of recommender systems include (1) demographic filtering (e.g., age, gender); (2) behavioral and content-based filtering (e.g., media use, length watched); (3) knowledge-based filtering (e.g., utilizes’ user feedback to provide recommendations); (4) social-based filtering (e.g., social network site connection); (5) general filtering (e.g., most viewed, editor’s choice; Hildén, 2022). An additional recommender system of interest is serendipitous filtering, which provides atypical recommendations leading to an unlikely viewing experience for the specific user (Oh et al., 2022). Netflix takes a unique approach in its recommender systems by utilizing a variety of algorithms that combine to create the Netflix homepage, which accounts for a reported “2 of every 3 h streamed” with an estimated value of one billion per year in revenue generated (Gomez-Uribe & Hunt, 2016, p. 2). Streaming services prioritize attracting or keeping users, not only through their content, but also through the organization and production of shows, which ultimately emphasizes the importance of algorithmic technology in user satisfaction (Pajkovic, 2022).

Both concerns (e.g., negates user choice, lack of transparency, company control) and benefits (e.g., increase quantity and diversity of content consumed, bringing attention to something unfamiliar) have been articulated with the increased utilization of algorithmic recommendations in streaming services (Khoo, 2023). For instance, the growth and rapid use of algorithmic recommender systems have led to questions about the relationship between algorithms and culture (Siles et al., 2019; Varela & Kaun, 2019). Rather than seeing this as a unidirectional relationship, algorithms not only shape our entertainment consumption and culture but are also cultural objects themselves, leading to a proposed bidirectional relationship (Khoo, 2023; Pajkovic, 2022; Siles et al., 2019; Varela & Kaun, 2019).

While there are obvious recommender tactics (e.g., *Because you watched*), companies also use “latent” tactics for recommendations that are less obvious. For example, Netflix asserts that, as users’ racial identities are not collected, identity markers are not built explicitly into the personalization experiences. Although refusals to include markers (i.e., “forbidden inputs”) may be seen as optimal practice, in truth, algorithms infer and utilize such demographic information to customize the marketing of content (Kearns & Roth, 2019). As a case in point, in 2018, Black Netflix customers expressed grievances with movie thumbnails displaying Black actors despite a minor role or a largely white cast in the movie (Eklund, 2022; Khoo, 2023; Meyerend, 2023). Hence, while Netflix’s decision not to include racial identity markers in its algorithms might be conceived as a form of “fairness,” in reality, algorithmic fairness is not met by the exclusion of information, because algorithms can make deductions from what is included to infer what is not included. Several scholars have raised awareness of and questions around structural concerns of racial discrimination and bias within algorithmic systems (Benjamin, 2019; Meyerend, 2023; Noble, 2018). Netflix’s thumbnails, perhaps, could constitute a form of “aesthetic harm” as they provided misleading information. Black Netflix users trusted the recommender systems, selected the recommendation, and discovered that this recommendation was not only inaccurate but was also suggestive of racial bias via the display of false, misleading thumbnails, thereby hampering users’ choice and their aesthetic experience.

Recommender systems are increasingly becoming more complex, intelligent, and agentic due to exponential advancements in machine learning and an unprecedented amount of data collected to provide recommendations for users (Kang & Lou, 2022). As algorithms are increasingly integrated into human social structures, such as mental health care (Ahmad et al., 2022), art (Ramesh et al., 2022), customer self-service (Nguyen et al., 2022), and loans (Kearns & Roth, 2019), agency is a growing interest in the study of artificial intelligence. In a recent Special Issue in the journal *Information Systems Frontiers*, Abedin et al. (2022) noted human-AI interactions are an understudied area, and emphasized the need to investigate the following topics: (1) AI user interface design; (2) human-AI conversations and collaboration; (3) explainability, accountability, ethics, fairness, and bias; and (4) AI agency and human-AI interaction with agentic AI. 

Explainability is the capacity for a machine-learning (or other AI) model to be adequately explained and reproducible (Abedin et al., 2022; Ehsan & Riedl, 2019; Shin, 2020, 2021; Varela & Kaun, 2019). Ehsan and Riedl (2019) emphasize the importance of meaning-making in this process and that “the explain- ‘ability’ of a computing system is often reliant on the human’s ability to make sense of its working. Thus, the meaning-making is a relational process where the alignment of situated epistemologies of the user and the machine needs to take place.”

While the explainability of machine-learning algorithms is typically oriented toward computer scientists, it should also be adapted towards communicating with novice end-users, thus needing different and accessible ways to explain their recommendations (Abedin et al., 2022). In one study, almost two-fifths of users reported not being aware that their favorite audiovisual streaming service (e.g., Netflix, YouTube) uses an AI recommender system (Chapman & Abraham, 2024a). Yet, users express a desire to understand how algorithms understand them (Choi et al., 2021). FATE (fairness, accountability, transparency, and explainability) acts as a heuristic in users’ trust, emotions, and satisfaction with algorithmic recommendations (Shin, 2021). Further work is therefore needed to understand if more thoroughly explained video recommendations increase the perceived quality and trustworthiness of an AI recommendation compared to a human recommendation (Kunkel et al., 2019). A question that has been less of a focus in these research domains is the potential for recommender systems to not only engage users but also shape and even change their aesthetic choices and sensibilities.

## 3. Aesthetic Choice

Choice and decision-making are multidisciplinary fields of study. Here, we specifically focus on aesthetically relevant choices. When examining algorithmic culture, understanding the nature and degree of influence that algorithmic recommender systems have on individual and societal aesthetic choice is necessary. Melchionne (2017) cautions that “the mere existence of algorithmic recommenders suggests that, at the core of our aesthetic lives, rests a choice problem crucial to aesthetic flourishing” (p. 286). He claimed that “aesthetic autonomy” is choice plus judgment, wherein choice is contingent, constructive, and part of a fulfilling aesthetic life. By contingency, he means that choice is influenced by a variety of contexts and conditions. A constructive approach implies a non-consistent method of choice, with the choice often being composed at the moment it is needed, thereby emphasizing the ephemeral nature of choice. Contingency and construction are balancing acts and may fluctuate depending on the circumstances. Melchionne (2017) emphasizes this by referencing a “preference for ice cream but only on warm days” (p. 289). In the case of audiovisual entertainment, the length of the content (e.g., movie vs. five-minute YouTube video) serves as a potential contextual factor that influences people’s decision to engage in a type of media (Chapman & Abraham, 2024a). Our aesthetic choices often involve leisure/entertainment (e.g., seeking pleasure), low risk (e.g., can stop watching a movie), incomplete information (e.g., wanting to know enough about a movie to be interested in it but not enough to ruin the suspense), and the unpredictability of creative agents (e.g., the surprise element of an artist and their artwork).

Since aesthetic choices are “low-stakes,” Melchionne (2017) conceptualizes that most aesthetic choices are made “informally” but that “good aesthetic choice requires noticing our responses to these opportunities” and is part of an *aesthetic plan* (p. 295). Aesthetic choice does not carry the same high-stakes decision-making that perhaps buying a house does. Instead, aesthetic choices become important through the accumulation of many choices, which contribute to our aesthetic sensibilities. The continuous, “small” choices create “patterns of experiences, zones of comfort, and horizons of curiosity… [and] lead to habits and expectations for attending to aesthetic objects” (Melchionne, 2017, p. 292). Drifting refers to these incremental, cumulative decisions. Drift choices describe how incremental choices eventually accumulate to important choices (Melchionne, 2017; Ullmann-Margalit, 2006).

In previous work, Melchionne (2015) outlined potential heuristics to cultivate aesthetic experiences including (a) avoiding overexposure to preferred objects (e.g., watching the same show repeatedly), (b) attending to the setting (e.g., not watching a movie in a busy or distractable environment), (c) appetite (e.g., choosing a show based on current mood rather than past enjoyment). Aesthetic plans consist of these drifting choices, which then align with a larger goal that is part of one’s aesthetic experiences (and thus life). Aesthetic plans promote awareness of judgment, create future aesthetic activities and engagements, and support the development of the person. Melchionne (2017) highlights the importance of lists as features of aesthetic plans, noting that “lists are at the center of the user interface of every streaming service suggest[ing] that list making is a core aesthetic practice” (p. 296). Ultimately, our aesthetic choices build towards a larger aesthetic plan.

An individual choice that requires a subjective interpretation, such as what to order at a restaurant or what to watch on Netflix, forces the person to balance preferences with uncertainty, through exploratory and exploitative behaviors (Riefer et al., 2017). Exploratory behaviors refer to seeking variation, risk-taking, and flexibility, whereas efficiency, refinement, quick returns, and low risk are classified as exploitative behaviors (e.g., using what is already known, such as buying the same product; Choi et al., 2021).

When an award is subjective (e.g., pleasure from food), Riefer et al. (2017) argue people conform to their choices through coherence maximizing, i.e., “the more people repetitively exploit an option the more entrenched their preference becomes” (p. 2). Upon a new discovery (e.g., a new restaurant), this process can start over and create new entrenchment processes that alleviate the burden of choice. Adamowicz and Swait (2013) proposed a two-stage model of consumer decision-making where a higher level of decision-making determines “how to make a decision” and the second stage designates “which product to purchase” (p. 18). The first stage emphasizes choice strategies prior to selection, where consumers choose based on three conventional choices: habitual, pure variety-seeking, or a fully evaluative choice. Accordingly, consumers choose either simple or complex cognitive processes to reach their selection based on current cognitive resources available (Adamowicz & Swait, 2013). Social influences impact people’s decision-making, where individuals utilize modeling as a form of choice. In modeling, an individual adapts their behavior to reflect other social actors (Higgs & Ruddock, 2020).

Individuals make continuous micro-level choices about food each day (Piqueras-Fiszman & Muñoz-Vilches, 2020), and their decision-making process is influenced by a variety of individual, contextual, and social factors. Contextually, time appears to play an important role in food choice. People tend to eat the same breakfast (an example of exploitive decision-making behavior) but display variety-seeking behaviors for lunches and dinners (Cadario & Morewedge, 2022). Ha and Jang (2013) found that the perceived quality of a dining atmosphere was related to the intention of choosing an alternative from familiar restaurants, whereas boredom was associated with either choosing an alternative from familiar restaurants or a new restaurant. Similar to the media literature, Cadario and Morewedge (2022) discuss hedonic and utilitarian goals that inform food-based decision-making.

Regarding choice and agency, user and machine agency negotiate energy trade-offs, in which the algorithms guide or clearly dictate their experiences on the streaming service (Kang & Lou, 2022). Sundar (2020) adapted and proposed the Theory of Interactive Media Effects (TIME) for the study of human-AI interaction. In the TIME model, recommender systems’ affordances (i.e., what they offer to the user) influence users’ engagement, perceptions, and trust through separate cue and action routes. When recommender systems are cued as a basis of user interactions, the user utilizes cognitive heuristics via shortcuts and prior perceptions of recommender systems, thus influencing perceptions of and responses to algorithmic recommenders in current use. In the action route, interaction, agency, social exchange, and mutual augmentation mediate user engagement with recommender systems. Human and AI recommender system collaboration then becomes an important feature of understanding the context of user experience and engagement with the platform of use. Therefore, algorithms and users collaborate, choose, and co-construct the media experience (Kang & Lou, 2022).

To recap, consumers utilize a variety of choice heuristics that are influenced by cognitive load, social context, goals, and individual factors. In this context, people’s repeated viewing (e.g., rewatching of the same show) of media must be acknowledged. Russell and Levy (2012) defined volitional reconsumption as “consumption experiences that consumers actively and consciously seek to experience again” (p. 341). A qualitative exploration of volitional reconsumption found consumers engage in reconsumption for emotional efficiency, self-existential understanding, and further insights into the reconsumption media (Russell & Levy, 2012). The concept of social surrogacy has been theorized to explain repeated viewings of a favorite television show, the idea being that repeated viewings are utilized for the ‘parasocial relationships’ developed with the fictional characters to cultivate a sense of belonging (Derrick et al., 2009). The relationship with characters is defined as parasocial in that viewers form attachments to the characters, but the characters are unable to reciprocate; it is a unidirectional relationship that mimics some aspects of a relationship but does not qualify as a fully formed, mutual relationship. People utilize repeated viewings of shows for a variety of reasons that ostensibly relate to well-being (e.g., connectedness, self-existential understanding). This suggests that people utilize repeated viewings of media may be linked to aesthetic flourishing, rather than a restriction of aesthetic interest. However, volitional reconsumption—which constitutes a clear demonstration of users exerting their aesthetic preference and choice—is an understudied area (Zemack-Rugar & Moore, 2019), and further studies are needed to provide a more nuanced understanding.

### 3.1. Entertainment Preferences

People’s entertainment preferences are incorporated into their aesthetic choices and plans. The Uses and Gratification theory proposes that consumers of media are active participants in their consumption of media content, which is influenced by motivations, past media gratifications, and audience activity (e.g., sharing favorite movies on social media) (Bondad-Brown et al., 2012). The theory emerged from the empirical studies of mass communication in the 1940s (Katz et al., 1973). Social and psychological needs function as catalysts for expectations and exposure to media, which have various consequences, such as gratifications gained from the media (Katz et al., 1974; Papacharissi, 2008). Along with social and psychological needs, technology may also influence one’s beliefs about media, that in turn impact people’s behaviors (e.g., intention to watch a movie) and their perceived gratifications (Palmgreen, 1984).

Emotions serve as a catalyst for types of gratification processes in entertainment experiences (Bartsch, 2012). Hedonic uses influence media preferences through emotional gratifications, such as mood management effects, wherein people seek movies to achieve a particular affective state (Vorderer & Halfmann, 2019). However, eudaimonic uses (i.e., a search for meaning, insight, and self-actualization) also drive entertainment selection and consumption (Reinecke & Kreling, 2022; Vorderer & Halfmann, 2019; Vorderer & Reinecke, 2015). This has led to a two-factor model being proposed that emphasized both pleasure-seeking (e.g., hedonic) and self-actualization (e.g., eudaimonic) as drivers of media consumption (Vorderer & Halfmann, 2019; Vorderer & Reinecke, 2015). Hedonism and eudaimonic effects better predict genre preferences than demographical factors like generational differences (Kim, 2020). Moreover, individuals who seek and move towards emotionally intense experiences were found to endorse more use of media for not only pleasure but also for eudaimonic motivations, and information, social interaction, and entertainment uses (Chapman & Abraham, 2024b).

Mere pleasure-seeking enjoyment is insufficient to describe eudaimonic motivations for entertainment engagement, as a movie that evokes sadness may lack enjoyment but contribute to meaning or reflection of the human condition (Oliver & Hartmann, 2010). Poignancy captures the mixed affect (i.e., co-occurring positive and negative emotions) that typifies a eudaimonic experience and gives rise to feelings of appreciation, which is a distinct type of enjoyment (Clayton et al., 2021). Oliver and Bartsch (2010) defined appreciation as “the perception of deeper meaning, the feeling of being moved, and the motivation to elaborate on thoughts and feelings inspired by the experience” (p. 76). Appreciation is largely conceptualized by means of two distinct approaches: (1) self-actualization (i.e., searching for meaning, truths, and purpose), and (2) self-determination theory (i.e., people’s daily need to satisfy a sense of autonomy, competence, and relatedness) (Ryan & Deci, 2000; Vorderer & Halfmann, 2019). People who have a desire for learning (i.e., intellectually curious) or are interested in learning about others (i.e., socially curious) report more eudaimonic motivations of media use (Chapman & Abraham, 2024b).

The inclusion of enjoyment and appreciation has supported development in self-transcendent media experiences that are distinguished by moral virtue, altruism, connection, and universalism with an emphasis on providing insight into the human condition through interconnectedness (Raney et al., 2018). Self-transcendent experiences (e.g., becoming aware of shared humanity) elicit specific emotions of awe, elevation, hope, and gratitude (Clayton et al., 2021). Self-transcendent emotions are assumed to relate to altruism, prosocial behaviors, and flourishing (Clayton et al., 2021; Raney et al., 2018). Chang (2023) proposed a model of media inspiration where viewers are inspired through three distinct processes—evocation (i.e., people are moved), transcendence (i.e., moving towards self-expansion), and motivation (i.e., viewers are motivated by media characters). In this model, inspiring media content comprises primarily three themes related to aesthetics: virtue, transformation, and creativity. Such proposals raise important considerations for considering broader transformation and creativity in self-transcendental media studies.

Taken together, people’s uses and motivations for entertainment media and engagement with media narratives widely vary and are influenced by a variety of contextual factors. While hedonic motivations prevailed as a dominant explanation, recent work has elucidated the broader aesthetic experience in contemporary media to include eudaimonic and self-transcendental media experiences. These experiences lead to interconnectedness, well-being, and support human flourishing (Chang, 2023; Clayton et al., 2021; Raney et al., 2018). The association between aesthetics, openness, and cognitive flexibility, among other concepts, suggests that aesthetic engagement is related to well-being.

### 3.2. Well-Being, Aesthetics, and Entertainment Media Engagement

Well-being is conceptualized as a multiple-dimensional construct that includes facets such as intellectual and emotional actualization, goals, connection, and contextual factors (Galderisi et al., 2015; Keyes, 2006). Hedonic well-being refers to a person’s happiness, life satisfaction, and balance of positive and negative affect (Diener et al., 2018), whereas eudaimonic well-being emphasizes self-actualization, development, positive evaluation of oneself, meaning-making, and positive interpersonal relationships (Keyes, 2006; Ryff & Singer, 2008). Though a full consideration of well-being is beyond the scope of this paper (for further consideration, see Diener et al., 2018; Galderisi et al., 2015; Keyes, 2006; Ryff & Singer, 2008), here, we are interested in the ways media, aesthetics, and technology influence people’s well-being. Since well-being includes facets of hedonia and eudaimonia, self-determination theory (SDT; Ryan & Deci, 2000) provides theoretical and empirical concepts to understand people’s motivations and uses in media and aesthetic engagement. SDT promotes three psychological needs that influence an individual’s motivations and well-being: (1) autonomy (i.e., moving towards one’s goals and values), (2) competence (i.e., challenges that lead to a sense of mastery), and (3) relatedness (i.e., experiences of connection and belonging; (Hutmacher & Appel, 2023; Peters et al., 2018; Ryan & Deci, 2000).

Regarding autonomy and mastery, certain personality characteristics are associated with aesthetic interests (e.g., openness to experience). Individuals who reported higher tendencies towards the aesthetic facet (e.g., being moved by art) in the openness trait were more likely to utilize learning from past experiences as a coping strategy (Johnson et al., 2023). Individual differences in the propensity for aesthetic chills (i.e., gathering goosebumps while listening to music) and aesthetic engagement were associated with stress-related growth orientation (i.e., conceptualizing stressful events as opportunities for growth; Johnson et al., 2023). Positive reinterpretation and growth, cognitive flexibility, and resiliency are hallmarks of stress-related growth orientation (i.e., “bouncing forward—not back”), which is relevant for well-being and differentiated from self-regulatory strategies like recovery or restoration (Johnson et al., 2023). Regulation and maintenance of aesthetic experiences are facilitated by working memory and concentration. This is because aesthetic appreciation requires a high demand of cognitive processing, schematic knowledge plays a role in art perception, and attentional mechanisms are utilized during an aesthetic response (Weigand & Jacobsen, 2022). Aesthetic savoring is hampered by stress and rumination, as those states interfere with the aesthetic processing mode (Weigand & Jacobsen, 2022). These different areas provide support for ways people’s aesthetic engagement and interest relate to their well-being and may connect to the importance of the cumulative effects (i.e., drifting) of aesthetic choices (Melchionne, 2017; Ullmann-Margalit, 2006).

Entertainment media may influence a person’s feelings of social connectedness. For example, people develop parasocial relationships with fictional characters that can support connection and reduce feelings of loneliness (Bond, 2021), complement social gratification (Gomillion et al., 2017), and support self-reflection (Oliver & Raney, 2011). Individuals may also use media to facilitate social interactions with others (Bartsch, 2012) or to supplement a lack of social interaction (Derrick et al., 2009). Algorithms play a role in the organization and curation of content, potentially creating a co-collaborative experience and interaction (Kang & Lou, 2022). As the digital entertainment landscape continues to evolve, the human–computer interaction field has encouraged designs “for deeper meaning, happiness, and human flourishing” (Peters et al., 2018). Hutmacher and Appel (2023) stated that “the operating characteristics of personalization algorithms can ultimately affect the satisfaction of the basic psychological needs and well-being” of the user (p. 29). Other proposed models situate people’s psychological needs as a mediator between the design and user outcomes, such as engagement and well-being (Peters et al., 2018). A recommender system’s perceived utility positively predicted the endorsement of the system and increases in users’ well-being (Chapman & Abraham, 2024a). In sum, the primary needs of autonomy, competence, and relatedness provide theoretical grounding to examine how digital media’s recommender systems potentially contribute to people’s well-being, whether through appreciation of aesthetics, stress-related growth orientations, parasocial relationships, or co-collaborative experiences.

## 4. Aesthetic Plans, Media Preferences, and Recommender Systems—A Collaborative Choice?

As discussed earlier, important concerns and potential harms have been emphasized with regard to the opaqueness with which algorithmic recommender systems function (Shin, 2020, 2021; Smuha, 2021). The impact on aesthetic choice has rarely been philosophically examined (Melchionne, 2017) and is scientifically largely unexplored (Khoo, 2023; Siles et al., 2019; Varela & Kaun, 2019). The multidisciplinary intersection of recommender systems’ influence on individual and social aesthetic choice is still in its infancy. This is a time of uncertainty when considering the impact of human-AI interaction on aesthetics, which makes it a fruitful period of inquiry for scientists and philosophers. In this section, we discuss the intersection of these fields and raise questions for future contemplation and research.

Melchionne (2017) provides heuristics for the consideration of aesthetic choices for aesthetic plans, such as avoiding overexposure to preferred objects. However, people select media for a variety of motivations and uses (Bondad-Brown et al., 2012). For example, volitional reconsumption supports emotional efficiency, self-existential understanding, and more insight into the specific media (Russell & Levy, 2012). Additionally, the food literature highlights the use of contextual influences (e.g., time of day; Cadario & Morewedge, 2022), which may also be applied to reconsumption. For instance, I may watch the same show when I am tired and desire parasocial comfort. The social surrogacy hypothesis (Derrick et al., 2009) supports this concept, namely that I form a para-relationship with the characters and feel comforted by the connection with them.

While Netflix claims people are “surprisingly bad at choosing between many options” (Gomez-Uribe & Hunt, 2016, p. 2), the choice literature describes a different story, where people utilize a variety of heuristics and models for consumer decision-making. Examples include coherency maximizing (Riefer et al., 2017), a two-stage model of consumer decision-making (Adamowicz & Swait, 2013), social influences (Higgs & Ruddock, 2020), contextual influences (Cadario & Morewedge, 2022), and goal-directed decision-making (Cadario & Morewedge, 2022). As Netflix’s recommender system accounts for roughly eighty percent of content watched on the platform (Gomez-Uribe & Hunt, 2016), it would seem almost improbable for it not to account for a significant portion of content watched, given that Netflix has an estimated 17,000 titles globally (Cook, 2022). How could one reasonably sort through the growing number of titles immediately available to them without the support of heuristics, or in this case, recommender systems? Even on the home screen, users are provided an abundance of recommendations, from which ultimately, they choose eighty percent of the time.

Netflix’s thumbnail images demonstrate users’ utilization of agency and choice in their aesthetic plan. Black Netflix users did not passively watch the falsely identified content that the recommender provided (Eklund, 2022; Khoo, 2023; Meyerend, 2023). Instead, they not only stopped consuming the recommender content but also voiced their displeasure (via social media) about the recommender’s aesthetic harm. The consumers utilized an agentic choice throughout this process. Recommender systems’ limitations to contextually draw inferences lead to an issue in relation to top–down recommendations. At least for long-form media, users do not indiscriminately follow algorithmic recommendations, as users expect the recommendation to coincide with their aesthetic plan. If a movie recommendation does not align with the user’s plan, the consumer may be more likely to discontinue watching the movie or select another option.

As Frey (2021) highlights, users reported following recommendations from a friend, family, or colleague most often while ranking the algorithmic recommender system at the bottom. This raises questions about what differentiates recommendations from algorithmic recommender systems compared to other types of recommenders (e.g., friends, professional critics). What cognitive, affective, or neural processes are activated when comparing recommendations from different systems? In other words, are different cognitive and affective processes activated when considering a recommendation from algorithms compared to a friend’s recommendation? Moving towards more “human-like” rationales, Ehsan and Riedl (2019) highlighted the need for recommender systems’ explainability to consider: (1) time and frequency of when the explanation is provided, (2) temporal evolution (e.g., changes over time of explanations), and (3) interaction paradigm (e.g., individual compared to group interactions with AI collaboration).

There are various open questions about the dynamics of recommender systems and human interaction. The human-AI interaction literature provides theory and models via fairness, accountability, transparency, and explainability (Shin, 2020) and the Theory of Interactive Media Effects (Sundar, 2020) to assess the relationships between recommender systems and humans’ aesthetic choices. (Kang & Lou, 2022) proposed exploring the collaborative relationship between algorithms and humans, which may positively influence people’s well-being through autonomy, connection, and mastery (Hutmacher & Appel, 2023; Peters et al., 2018). It would be worthwhile to also investigate the relationship between recommender systems, aesthetic choices and plans, and users’ well-being.

## 5. Cultivation of Aesthetic Sensibilities

As Melchionne (2017) suggests, our aesthetic sensibilities are cultivated through the accumulation of various aesthetic choices. While he provides a few heuristics to support the cultivation of aesthetic choices, more focus is needed to understand the various ways aesthetic sensibilities can be cultivated through a plan of aesthetic choices. Doddington (2021) contends that meaningfulness relies on embodied, aesthetic experiences and provides grounds for human flourishing, and argues for schools to include aesthetics in the classroom as it creates shared experiences that bolster trust, meaning, and transformation of students’ perceptions. D’Olimpio (2022) defends a similar proposition, claiming that a flourishing life includes aesthetic appreciation and experiences. Given that around 40 percent of people were not aware that their favorite streaming service uses AI-recommender systems (Chapman & Abraham, 2024a), and in line with the need to have explainability and transparency, aesthetic education (akin to an informed consent process) about recommender systems may contribute to the understanding of recommender systems.

Since recommender systems are integrated into our daily lives, we need to understand how people interact and collaborate with them to assess and support aesthetic sensibilities. Future work can focus on educating people about ways in which they interact with recommender systems, the potential influence on their choices, and ultimately, their well-being. Educating novice users in their understanding of recommender systems addresses important findings (e.g., AI agency, explainability, bias; Abedin et al., 2022). Aesthetic experience connects us to ourselves and to others. Recommender systems are part of our connection to audiovisual entertainment media, and thus, collaborators in our aesthetic plan. Education will assist us in understanding the limitations, concerns, strengths, and support that recommender systems provide to us. If we are to support aesthetic plans, then promoting people’s understanding of recommender systems through the educational system is important.

Continued evidence is also needed to understand how people cultivate their aesthetic sensibility in practice, as well as the influence of aesthetic choices on other aspects of well-being, flourishing, and mental health. Additionally, we need to consider the role recommender systems play in a person’s aesthetic plan and whether they can assist in cultivating a person’s aesthetic sensibility. Educating people to utilize the recommender systems as collaborators in their aesthetic perhaps promotes opportunities to utilize the available resources. Since self-transcendental media is about interconnectedness (Clayton et al., 2021), perhaps we need to consider our interconnectedness with recommender systems. Viewers build parasocial relationships with characters in media; do viewers build relationships with recommender systems as well?

From these different fields, we outline several potential questions to explore.

- Aesthetic choice:
  ○ Does creating and attending to an aesthetic plan over time help a person develop their sense of aesthetic sensibility and support their well-being?
  ○ How do contextual constraints (e.g., time), social factors (e.g., watching a movie with a partner or friends), and individual factors (e.g., openness) influence aesthetic choices?
- Aesthetic education
  ○ How can the education of recommender systems, aesthetics, and modern media influence the perception and collaboration with recommender systems?
- Algorithmic recommender systems
  ○ Do algorithmic recommender systems restrict or cultivate aesthetic choices and support plans, and if so, how?
  ○ Would user content consumption differ between full control of choice in selection compared to an AI-curated selection of media?
  ○ What role does perceived agency from algorithmic recommender systems play in influencing the use and satisfaction of aesthetic choices?
  ○ What is the impact of obvious compared to more latent recommendations on aesthetic choices? How aware are people of the differences, and does it lead to different types of engagement with the streaming platform and media engagement?
- Cross-cultural impacts of algorithms
  ○ In what ways do cultural differences influence the acceptance and trust of algorithmic recommendations?
  ○ Do cultural differences influence people’s aesthetic choices, and what interaction do they have with algorithmic recommendations?

## 6. Conclusions

Over the last few decades, we have significantly changed the way we engage with modern entertainment media. We are inundated with entertainment options, and to support our viewing experiences, companies utilize a variety of machine-learning algorithms to provide personalized recommendations. The increased integration of AI algorithmic recommender systems in our entertainment media consumption has led to concerns and uncertainty about the impact on our aesthetic choices and overall culture (Khoo, 2023; Melchionne, 2017; Siles et al., 2019; Varela & Kaun, 2019). Nonetheless, the choice and decision-making literature demonstrate a variety of different approaches to choice, whether that is their own heuristic model of decision-making or a family member’s recommendation (Frey, 2021), and are influenced by cognitive load, social context, goals, and individual factors (Adamowicz & Swait, 2013; Cadario & Morewedge, 2022; Ha & Jang, 2013; Riefer et al., 2017). Thus, people’s utilization of algorithmic recommender systems does not imply poor decision-making. Instead, it is a tool for more efficient decision-making. Our time is a limited resource; certainly, we could navigate through the thousands of movies and show titles on a streaming platform, but is that feasible for people to do regularly?

Recommender systems’ influence on aesthetic choices, plans, and sensibility requires further consideration. We need to investigate the spectrum of potential benefits (e.g., supporting choice in service of aesthetic plan) and potential harms (e.g., restricting or skewing choice in disservice to aesthetic plan). The human-AI interaction literature provides interesting and relevant considerations for future studies (Shin, 2020; Sundar, 2020), and for human and AI collaboration (Kang & Lou, 2022; Sundar, 2020). Future studies need to consider the various individual, contextual, cultural, and social factors that influence people’s relationships with streaming services recommender systems. Educating individuals on their relationship with and to technology (and in this case recommender systems) appears to be an important area of focus. Our judgments about the influence of recommender systems on our aesthetic choices need to be reserved until more evidence is made available.

## Data Availability

No new data were created or analyzed in this study.

## Note

1 It is also important to state *what this paper is not*. This paper does not aim to discuss or make claims about the ethical use of AI recommender systems. While the paper discusses inequities, the primary purpose is not to discuss the various ethical harms of algorithmic recommender systems. The paper does not make a claim about AI-designed art. The paper focuses on algorithmic recommender systems and aesthetic experiences, and we approach it from an interdisciplinary perspective. For readers interested in AI ethics or harm (see Benjamin, 2019; Eklund, 2022; Kearns & Roth, 2019; Khoo, 2023; Meyerend, 2023; Noble, 2018).
